# A Patient Simulation Framework for Risk Assessment of Conversational Health Care AI: Development and Evaluation Study

**DOI:** 10.2196/100772

**Published:** 2026-10-05

**Authors:** Md Tanvir Rouf Shawon, Mohammad Sabik Irbaz, Hadeel R A Elyazori, Keerti Reddy Resapu, Yili Lin, Vladimir Franzuela Cardenas, K Pierre Eklou, Farrokh Alemi, Kevin Lybarger

**Affiliations:** 1 Department of Computer Science, College of Engineering and Computing, George Mason University, Nguyen Engineering Building, 4511 Patriot Cir, Fairfax, VA, 22032, United States, 1 571-663-5183; 2 Department of Information Sciences and Technology, College of Engineering and Computing, George Mason University, Fairfax, VA, United States; 3 Department of Health Administration and Policy, College of Public Health, George Mason University, Fairfax, VA, United States; 4 School of Nursing, College of Public Health, George Mason University, Fairfax, VA, United States

**Keywords:** natural language generation, consumer health, health informatics, patient simulation, conversational agents, AI risk management, trustworthy AI, large language models, depression management

## Abstract

**Background:**

Conversational AI systems are increasingly being deployed in health care for clinical decision support, but their performance varies substantially across patient communication styles, health literacy levels, and behavioral patterns. Static benchmarks cannot capture multiturn dynamics through which this variation compounds, and no current evaluation framework implements structured AI risk management guidance for conversational health care AI. The result is a structural risk: AI systems may perform well in aggregate while failing disproportionately for the populations they are intended to help.

**Objective:**

This study aimed to develop and validate a patient simulation framework that aligns with the National Institute of Standards and Technology (NIST) AI Risk Management Framework (AI RMF) Map and Measure functions, providing an empirical basis for identifying and characterizing performance risks in conversational clinical AI across medical, linguistic, and behavioral patient variations. We applied the framework to a conversational decision aid for antidepressant selection in major depressive disorder (the AI decision aid).

**Methods:**

The simulator integrated three profile dimensions: (1) medical profiles constructed from All of Us electronic health records using risk ratio gating; (2) linguistic profiles modeling a health literacy gradient and condition-specific communication; and (3) behavioral profiles representing cooperative, distracted, and adversarial engagement. We generated 500 simulated conversations and evaluated profile fidelity through human annotation and a large language model (LLM) judge, and then assessed downstream effects on the AI decision aid’s concept retrieval and antidepressant recommendations.

**Results:**

The patient simulator expressed medical concepts with high fidelity (96.6% accuracy across 8210 concepts), with substantial human interannotator agreement (κ=0.73) and LLM-judge agreement against human annotators (κ=0.78). Behavioral profiles were reliably distinguished (κ=0.93; near perfect agreement), and linguistic profiles showed substantial agreement at the lower bound of the substantial range (κ=0.61), which can be considered adequate to support profile-level analysis. The framework revealed monotonic degradation in AI decision aid performance across the health literacy gradient. Rank-1 concept retrieval increased from 47.6% for limited health literacy to 81.9% for proficient health literacy, with corresponding declines in antidepressant recommendation accuracy.

**Conclusions:**

Patient simulation grounded in the NIST AI RMF exposes measurable performance risks in conversational health care AI that static benchmarks miss, with direct equity implications. Health literacy operates as a structural risk factor, with degraded performance concentrated in patients carrying the greatest burden of psychiatric illness. The framework supports targeted risk-mitigation interventions before deployment. While we evaluated the framework only on antidepressant selection, extending it to other clinical decision-aid tasks remains an assignment for future work.

## Introduction

### Background

Conversational AI systems are being increasingly deployed in health care to support clinical decision-making, patient engagement, and care accessibility [1-4]. Yet these systems face a fundamental evaluation gap: patients communicate the same clinical information in vastly different ways depending on health literacy, psychological state, and interaction style [5,6]. A patient who says, “my morning pill for my nerves,” and another who reports, “20 mg of fluoxetine for generalized anxiety disorder,” may describe identical clinical realities, but a conversational agent that handles the second statement better than the first introduces a structural bias against vulnerable populations. Recent work demonstrates that large language model (LLM) clinical outputs shift measurably when patient messages are perturbed with stylistic, syntactic, or demographic variation, with disparities concentrated in vulnerable subgroups and amplified in conversational settings [7]. There is no standardized method to assess whether AI performance remains equitable across this variation [8,9], so disparities are likely to surface only after deployment, with disproportionate impact on populations facing existing barriers to equitable care. LLM-based approaches lower barriers to building such systems [10,11] but heighten the need for rigorous, scalable risk assessment that can characterize performance across diverse patient communication patterns.

Static benchmarks cannot capture these risks because they bypass the multiturn dynamics through which communication variation compounds [12,13]. Patient simulation offers a scalable alternative: simulated patients with controlled clinical, linguistic, and behavioral characteristics can systematically probe system performance across populations that would be difficult, slow, or ethically complex to recruit for live evaluation [14]. Simulation-based methods are also being used more broadly to quantify LLM risk in health care, for example, to estimate hazard-to-harm probabilities for regulatory risk assessment of LLM-based medical devices [15]. What is missing is a structured framework for connecting patient simulation to AI risk governance. The National Institute of Standards and Technology (NIST) AI Risk Management Framework (AI RMF) provides domain-agnostic guidance for identifying, assessing, and managing AI risks [16], but no existing patient simulation framework operationalizes this guidance for conversational health care AI [8,9]. This work introduces such a framework. Patient simulator construction supports the Map function by structuring risk identification across medical, linguistic, and behavioral profile dimensions. Simulator-AI interaction supports the Measure function by surfacing performance variation across the structured profile space. The framework surfaces the evidence needed for AI RMF–aligned risk analysis. Extending this evidence into deployment-specific risk analyses is left for future work.

We present a patient simulator grounded in real-world clinical data from the All of Us Research Program [17], evaluated on a conversational decision aid for antidepressant selection in major depressive disorder. The simulator integrates three profile dimensions: (1) medical profiles constructed from electronic health record (EHR) data through a risk ratio (RR)–based feature selection process that prioritizes outcome-relevant clinical features while preserving statistical independence and clinical coherence; (2) linguistic profiles modeling health literacy variation [18,19] and condition-specific communication patterns [20,21]; and (3) behavioral profiles representing empirically derived interaction patterns, including cooperative, distracted, and adversarial engagement [22,23]. The framework generalizes across health care tasks and conditions. This study evaluates it on antidepressant selection while also addressing the following research questions (RQs):

- RQ1: How effectively can structured EHR data be transformed into risk-aware medical profiles aligned with AI trustworthiness principles?
- RQ2: How can simulated patients combining medical, linguistic, and behavioral information produce realistic and distinguishable conversational behavior?
- RQ3: How does simulated patient variation across medical, linguistic, and behavioral profiles affect the performance of a conversational clinical decision aid, and which patient subgroups are most affected?

This work provides the following: (1) a patient simulation framework that aligns with the NIST AI RMF Map and Measure functions for conversational health care AI, integrating medical, linguistic, and behavioral profiles; (2) empirical evidence that health literacy creates a monotonic performance gradient in conversational clinical AI, identifying a concrete equity risk; (3) a RR-based algorithm for generating outcome-relevant, auditable medical profiles from EHR data; and (4) a controlled perturbation methodology using ontology-aware semantic substitution for validating both human annotators and LLM judges. The work was conducted by a multidisciplinary team integrating clinical psychiatric and mental health nursing expertise, clinical informatics, and natural language processing, with guidance from a patient and clinician advisory board. The code, source data, and simulated interactions are publicly available [24].

### Related Work

#### Overview

In this section, we review related work on user simulation, patient simulation, and behavioral and linguistic modeling for conversational AI. User simulation provides scalable methods for evaluating conversational agents across domains, and patient simulation adapts these methods for clinical dialogue and diagnostic reasoning.

#### User Simulation

User simulation enables controlled, scalable evaluation of interactive systems without requiring human trials. Foundational surveys span information access, dialogue modeling, and recommendation systems [14,25-27], while recent work leverages LLMs to generate context-aware user behavior through dual-model architectures combining generators and verifiers [28,29] and session-level search simulation [30,31]. In health care, synthetic users with clinical profiles evaluate decision support and coaching systems [32], establishing user simulation as a critical method for assessing reliability and safety in AI systems. Mental health conversational AI research has historically split between technical evaluation of response quality and clinical evaluation of patient outcomes, with limited integration across the two [33].

#### Patient Simulation

Patient simulation involves the construction of virtual patients that engage in interactive clinical dialogues with human or automated clinicians [34]. Frameworks vary widely in patient state representation, from hand-crafted profiles to EHR-grounded models, and in how they control disclosure, tone, and clinical accuracy [1,35,36]. Prior work has often treated patient simulation as a single unified problem, but it involves two distinct technical challenges: (1) constructing clinically valid patient representations and (2) simulating interactive dialogue that expresses those representations over time [36]. Recent systems have integrated LLMs to enhance expressiveness and flexibility [37], constructed structured knowledge bases from clinical notes to support medical intake tasks [38], and embedded risk-aware feedback mechanisms [34].

#### Data-Driven Medical Realism

Early systems used deterministic state machines or probabilistic sequence models to generate medical histories. Synthea [39] and SynSys [40] produce scalable, transparent timelines but reflect population-level distributions rather than individual patient data. EHR-grounded approaches improve realism. Generative Adversarial Network (GAN) variants [41,42] address missing features and mixed data types, achieving higher fidelity than rule-based methods on MIMIC-III/IV benchmarks [43], while SimSUM [44] combines Bayesian networks with prompted LLMs to synthesize clinical notes. Systems integrating EHR grounding with conversational retrieval, including ophthalmology simulators [38] and multiagent knowledge graph pipelines [45], advance clinical accuracy but treat the encounter as a transactional data exchange rather than a dynamic, rapport-dependent interaction.

#### Behavioral and Linguistic Modeling

Beyond medical profile construction, systems must express profiles through natural dialogue with appropriate communication style and conversational dynamics [35,46]. Cognitive and persona-based approaches model psychological states to generate behaviorally coherent conversation. PATIENT Ψ [47] uses expert-designed cognitive schemas with GPT-4 to reproduce emotional fluctuations and resistant behaviors, while SFMSS [48] embeds Big Five traits to shape dialogue tone and coordinates patient, nurse, and supervisor agents to enforce outpatient triage workflows. PAL [49] simulates emotionally nuanced palliative care patient interactions with NURSE-framework feedback, illustrating affect-grounded simulation in another condition-specific clinical context. These systems remain condition-specific (eg, mental health and palliative care) and lack medical grounding for clinical evaluation. Prompt-based approaches [34,37] offer scalability but lack persistent patient state tracking and systematic control over linguistic and behavioral variation.

#### Procedural and Workflow Control

A third class prioritizes procedural fidelity and clinical workflows through discrete agent roles and actions. iPDG [50] enforces clinical plausibility through manually defined domain rules. Such systems achieve procedural coherence but sacrifice conversational flexibility and behavioral depth.

#### Limitations and Gaps

Recent systematic reviews of mental health chatbots found that LLM-based systems concentrate in early stage technical validation, with most evaluations focused on conversational quality or adherence to specific prompts in controlled settings rather than rigorous testing for clinical benefit [51]. Existing patient simulation systems exhibit 2 critical gaps that constrain trustworthy AI evaluation. First, no existing framework integrates medical realism, behavioral variation, and linguistic diversity within a unified architecture. Recent systems pair medical grounding with behavioral modeling or behavioral depth with linguistic variation, but this fragmentation creates evaluation blind spots. Existing systems cannot systematically test whether agents maintain diagnostic accuracy across complex comorbidities, varied health literacy, and adversarial behavior simultaneously. Multiturn failures like context loss and inconsistent safety emerge precisely at these intersections.

Second, existing approaches prioritize medical outcomes over systematic risk management aligned with frameworks like the NIST AI RMF. They lack explicit risk mapping connecting simulation parameters to risk categories, auditability with traceable lineage to source data, and controllable risk probing through systematic variation. This prevents the detection of consequential failures: agents may recommend different treatments when patients express identical conditions at different health literacy levels, or safety mechanisms may fail under adversarial conditions that only systematic variation can expose. We address both gaps through a unified framework whose medical, linguistic, and behavioral profiles align with the NIST AI RMF Map and Measure functions, enabling risk assessment across medical accuracy, communication appropriateness, and behavioral robustness.

## Methods

### Overview

The framework integrated a patient simulator with an AI decision aid to enable systematic evaluation of conversational clinical decision-making. The patient simulator produced controlled, profile-driven responses by combining medical, linguistic, and behavioral characteristics, while the AI decision aid conducted a structured conversational intake to elicit clinical history and generate antidepressant recommendations. Figure 1 illustrates this interaction. This section details the patient simulator design, AI decision aid, and evaluation methodology.

**Figure 1.**
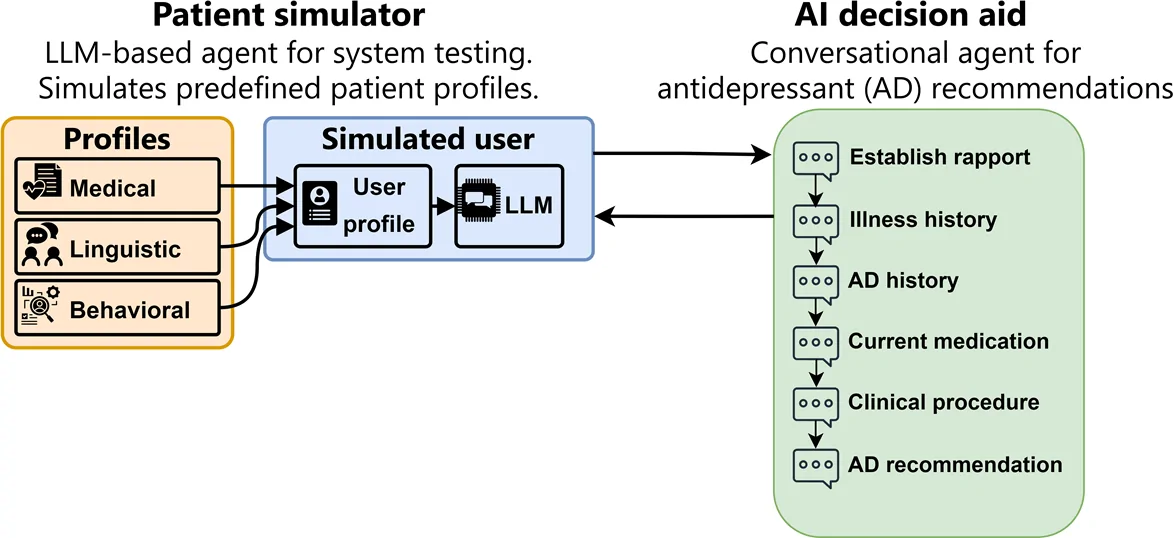
A schematic diagram of the conversation between the patient simulator and the AI decision aid system. LLM: large language model.

The research team integrated clinical expertise in psychiatric and mental health nursing with expertise in clinical informatics, health administration, and natural language processing, with clinical authors contributing to the patient profiles, antidepressant selection task, and evaluation framework. The broader Patient-Centered Outcomes Research Institute (PCORI)–funded project was guided by an advisory board of clinicians, mental health organization leaders, and individuals with lived experience of depression.

### Data

#### Overview

Both the patient simulator medical profiles and the AI decision aid prediction models were derived from the All of Us Research Program Registered Tier v8 dataset, a national longitudinal study providing structured EHR data, including conditions, medications, procedures, and demographics [17].

#### Cohort Selection

We extracted data from All of Us participants with major depressive disorder (Systematized Nomenclature of Medicine Clinical Terms [SNOMED CT] code 370143000 and its descendants). The resulting cohort included 58,446 participants, contributing 466,752 antidepressant trials, with 18,471 diagnoses, 2642 medications, and 5001 procedures. Outcomes captured responses to 14 antidepressants and 1 category covering all remaining antidepressants (dataset cutoff: October 1, 2023). Antidepressant response was defined as taking the antidepressant for at least 10 weeks without switching or augmenting with another antidepressant [52].

#### Data Application

For patient simulator medical profiles, the dataset provided feature distributions, RR calculations for outcome-relevant predictor selection, and demographic distributions for age and gender initialization. The simulator was evaluated on the 4 most common antidepressants: fluoxetine, sertraline, trazodone, and duloxetine [53]. For the AI decision aid, the same data trained prediction models generating treatment recommendations across all 14 antidepressants. All data use complied with All of Us dissemination policies.

### Patient Simulator Design

#### Design Rationale and NIST Alignment

The patient simulator design was grounded in the NIST AI RMF, which defines four core functions: (1) *Govern* (accountability and oversight), (2) *Map* (risk identification), (3) *Measure* (risk assessment), and (4) *Manage* (risk mitigation) [16]. We aligned with Map and Measure across 2 complementary phases. Simulator construction supported Map by structuring risk identification through 3 profile dimensions, each addressing a distinct category of risk: medical profiles targeting clinical accuracy, linguistic profiles targeting communication-dependent risks, and behavioral profiles targeting interaction-dependent risks. Simulator-AI interaction supported Measure by surfacing how AI performance varies across that structured profile space, facilitating downstream risk characterization.

#### Medical Profile Requirements

Medical profiles provided the clinical foundation for risk assessment, requiring realistic and diverse medical contexts to evaluate performance across clinical heterogeneity. Generating such profiles from structured EHR data faces interconnected challenges: high-dimensional feature spaces (10⁵-10⁶ concept codes) with sparse task-relevant signals, data quality issues (missingness and inconsistent coding), and medically implausible feature combinations [54-56]. Our design adhered to five AI RMF–aligned trustworthiness requirements: (1) *controllability* (emphasize task-relevant features while maintaining outcome diversity), (2) *coherence* (maintain clinical plausibility across diagnoses, treatments, and temporal events, including rare but valid scenarios), (3) *variability* (capture heterogeneous comorbidities and contextual factors to expose brittleness and subgroup bias), (4) *efficiency* (balance clinical completeness with computational tractability for large-scale evaluation), and (5) *transparency* (maintain traceable feature lineage to source distributions for targeted risk analysis). The mapping of these requirements to data challenges and mitigated risks is provided in Multimedia Appendix 1.

#### Linguistic and Behavioral Profile Requirements

Medical profiles establish clinical context but cannot capture communication-dependent and interaction-dependent risks, requiring 2 additional profile dimensions, consistent with the safety taxonomy proposed by Lim et al [57]. These are needed because accurate medical retrieval can still fail due to how a patient communicates or how the conversation unfolds. The 2 dimensions map onto the patient input types and hazardous scenarios mentioned by Lim et al [57], informing the communication-dependent and interaction-dependent categories used here.

Communication-dependent risks emerge from heterogeneity in patient expression. Agents must maintain safety and comprehensibility across health literacy levels, condition-specific patterns, and vernacular variations [58]. Linguistic profiles enable controlled assessment grounded in health literacy and psycholinguistic research [59].

Interaction-dependent risks emerge when patients manipulate information, test boundaries, or withhold details [60]. Because evaluation cannot assess safety mechanism robustness without systematic behavioral variation, behavioral profiles are grounded in clinical and human-computer interaction research to probe agent resilience across conditions.

#### Implementation

The patient simulator integrated 3 profiles: medical profiles grounded in EHR data, linguistic profiles capturing health literacy and condition-specific variation, and behavioral profiles representing engagement and interaction patterns. Profiles were operationalized through structured LLM prompting, where the medical profile assigned hierarchical indices to each clinical fact (eg, [3.2] Individual Psychotherapy). For each intake question, the model identified relevant indexed facts; applied linguistic style transfer (eg, [3.2] “talked to someone”); and constructed a JSON response containing the original facts, style-transferred equivalents, and a final natural language response with inline span markers. Simulator-provided markers were removed before being passed to the AI decision aid to ensure independent extraction. The complete prompt is provided in Multimedia Appendix 1.

#### Medical Profiles

Phase 1 generated outcome-relevant, coherent, and diverse patient profiles. Phase 2 applied probabilistic selection using a binomial distribution derived from All of Us antidepressant response data, ensuring that the final cohort reflects realistic population-level variability.

### Medical Profile Generation (Phase 1)

#### Overview

Profile generation used three core design principles [61,62]: (1) prioritize outcome-relevant features, (2) enforce statistical independence among selected features, and (3) inject controlled diversity from residual features, implemented through 4 stages (relevance filtering, demographic initialization, independence screening, and diversity expansion), as outlined in Figure 2.

**Figure 2.**
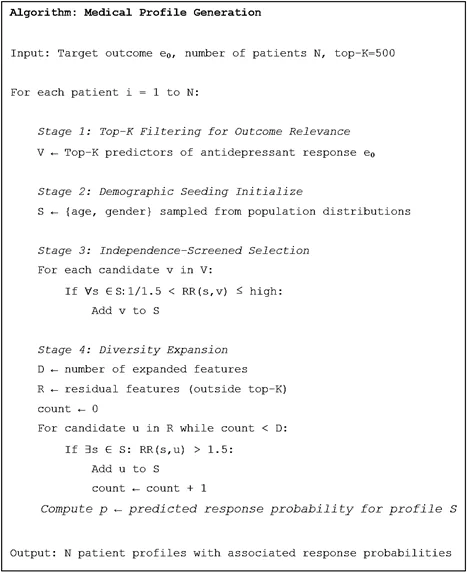
Abbreviated medical profile generation algorithm. Complete algorithm details are provided in Multimedia Appendix 2.

#### Stage 1: Top-K Filtering

The algorithm restricted feature candidates to the top K predictors of the antidepressant response outcome, *e*_0_ (K=500), improving sample efficiency and aligning with the max-relevance component of minimum redundancy maximum relevance feature selection [62].

#### Stage 2: Demographic Seeding

Each profile *S* was initialized with age and gender from All of Us demographic distributions.

#### Stage 3: Independence-Screened Selection

Features were added iteratively, with each candidate *v* screened for statistical independence from already-selected features. The RR *RR*(*s*,*v*) quantifies the association between features *s* and *v* with respect to antidepressant response:


$$RR(s,v)=\frac{P(response\;|\;s\;\cap \;v)}{P(response\;|\;s)}$$

where values near 1 indicate independence, values >1.5 indicate positive association, and values <0.67 indicate negative association. Each candidate was required to satisfy:


1/1.5<RR(s,v)≤high,∀s∈S

This symmetric band excluded near-deterministic couplings (*RR* >high) and strong anticorrelations (*RR* <1/1.5), ensuring statistically independent and clinically coherent feature combinations [61,62]. The upper threshold (high) was set to 7.

#### Stage 4: Diversity Expansion

After forming a coherent feature set, residual features outside the top-K set were added if they satisfied:


RR(s,u)>1.5∃s∈S

This captured latent contextual variables enriching patient heterogeneity without compromising coherence [61,62]. The complete pseudocode is provided in Multimedia Appendix 2.

### Probabilistic Patient Selection (Phase 2)

#### Overview

Phase 2 selected patients whose response probabilities reproduce the All of Us population-level distribution by dividing the probability range into 7 sigma bands of a binomial distribution *B*(*n*,*p*), with sampling weighted to match expected binomial frequencies. For example, with *n*=100 and *p*=0.4 (*μ*=40; *σ*≈4.9), approximately 64% of patients fall within (*μ*-*σ*, *μ*+*σ*), 15% in each adjacent band (*μ*±1*σ* to *μ*±2*σ*), and <3% in the tails beyond *μ*±2*σ*. This stratified sampling preserves central tendency and natural variability while avoiding over- or underrepresentation of extreme responders. Detailed sigma-band allocations appear in Multimedia Appendix 2.

For AI RMF alignment, *controllability* and *coherence* were achieved through outcome-relevant feature selection and RR screening, captured by the proportion of outcome-related features and the average RR. *Variability* was assessed via the number of unique concept codes, *efficiency* was determined by the average features per profile, and *transparency* was evaluated through explicit feature lineage enabling auditable profile construction.

The framework had 2 tunable hyperparameters governing profile density: the high threshold from stage 3 and the number of residual features added in stage 4 (set here to 3‐5 per profile). Lowering *high* narrowed the set of candidate concepts eligible for inclusion. Varying it across {4,5,6} yielded 5‐8 features per profile on average versus 8‐9 at *high*=7, a gradual rather than sharp change indicating limited sensitivity to the exact threshold within this range. Either hyperparameter can be adjusted to target a desired profile density.

#### Linguistic Profiles

Conversational agents must maintain safety and comprehensibility across diverse patient expression styles [63,64]. Systematic linguistic variation exposes blind spots and failure modes [65,66]. The simulator implemented a dual-axis linguistic framework with profiles along two independent dimensions: (1) a health literacy gradient capturing variation in comprehension, terminology, and discourse structure [19,59,67,68], and (2) condition-specific communication reflecting linguistic patterns characteristic of depression and anxiety disorders derived from Linguistic Inquiry and Word Count (LIWC)–based clinical analyses [69]. Health literacy generalizes across clinical tasks as comprehension barriers affect patient-agent interaction regardless of medical condition. Condition-specific profiles capture diagnostic patterns (*depression* and *anxiety*) adaptable to other conditions. Table 1 specifies the 5 linguistic profiles.

The linguistic profiles align with NIST requirements through literature-grounded linguistic variation [19,59,67-69], enabling controllable assessment of communication-dependent risks.

**Table 1.** Linguistic user profiles across multiple dimensions.

Profile	Key linguistic attributes	Example response
Health literacy		
Limited	Style: Concrete, informal, sometimes vague Tone: Hesitant, uncertain, conversational Vocab: Everyday terms, slang, vague quantities Structure: Short, fragmented sentences; frequent fillers Patterns: Minimal elaboration unless prompted	“Uh, just my morning pill. you know, the one for my nerves.”
Functional	Style: Clear, basic descriptions of symptoms or routines Tone: Cooperative, open Vocab: Mix of common and medical terms Structure: Simple narratives; occasional causal reasoning Patterns: Provides coherent answers; asks clarifying questions	“I take Prozac every morning. It helps my mood, but I still have trouble sleeping.”
Proficient	Style: Precise, clinical, well-organized Tone*:* Confident, analytical Vocab: Technical terms; qualifiers such as “likely” or “seems improved” Structure: Multiclause, logically sequenced sentences Patterns*:* References timelines; anticipates follow-up questions	“I’m on fluoxetine, 20 milligrams daily. It’s effective, though I’ve noticed mild insomnia.”
Condition-specific		
Depression	Style: Brief, muted, sometimes resigned Tone: Flat, pessimistic, self-critical Vocab: Negative emotion words; self-focused phrasing Structure: Short, often past-tense statements Patterns: Withdrawn responses; dismisses reassurance	“Barely sleeping. My head won’t shut off.”
Illness anxiety disorder	Style: Symptom-focused and repetitive Tone: Anxious, urgent Vocab: Symptom terms; “what if” speculation; absolutist wording Structure: Mix of run-on sentences and abrupt alarms Patterns*:* Reassurance-seeking cycles; future-oriented worry	“I felt a flutter. What if it’s heart failure even though the test was normal?”

#### Behavioral Profiles

Conversational agents struggle when patients go off-topic, respond vaguely, or withhold information [70,71]. Systematic behavioral variation is essential for stress-testing agent robustness and assessing recovery from conversational breakdowns [72,73]. We organized the 13 patient behaviors documented by Simpson et al [74] into 4 behavioral categories (complete mapping is provided in Multimedia Appendix 2), with *structured and cooperative* as an additional fifth category. This study operationalized 3 profiles: *distracted and unfocused* and *adversarial and combative* to capture challenging dynamics, with *structured and cooperative* as the baseline. We selected this subset to bound experimental scope while preserving the contrast that matters for risk assessment: a cooperative baseline against 2 profiles that diverge in distinct ways. Each profile varied along 4 dimensions: conversational adherence, engagement, topical focus, and adversarial behavior, as detailed in Table 2.

**Table 2.** Behavioral user profiles across multiple dimensions.

Profile	Key attributes	Example response
Structured and cooperative	Adherence: High Engagement: High Topical focus: High Adversarial/toxic behavior: Minimal	“Yes, I take 20 mg of fluoxetine every morning around 8 AM. I haven’t missed a dose in the last three weeks.”
Distracted and unfocused	Adherence: Low Engagement*:* Sporadic Topical focus*:* Off-topic Adversarial/toxic behavior*:* Inadvertent derailment of conversation	“I was. wait, which one? Oh right, yeah I think? But yesterday I forgot — also my dog wouldn’t eat.”
Adversarial and combative	Adherence*:* Variable Engagement*:* Variable Topical focus*:* Variable Adversarial/toxic behavior: Overtly confrontational or hostile	“What kind of dumb question is that? Maybe if your system worked better, I wouldn’t have to answer this again.”

The behavioral profiles align with NIST requirements through empirically grounded behavioral variation [74], enabling controlled assessment of interaction-dependent risks including adversarial scenarios with transparent lineage.

Together, these 3 profile dimensions provide comprehensive Map and Measure coverage for conversational health care AI risk assessment.

### Chain-of-Thought Prompting Strategy

We used chain-of-thought (CoT) prompting [75] to generate realistic patient responses through a structured process. For each question posed by the AI decision aid, the prompt directed the model to (1) identify relevant indexed medical attributes, (2) apply controlled term-level linguistic transformations, and (3) construct a natural language response with explicit references. Behavioral constraints were enforced as rules governing interaction, while linguistic constraints shaped the form of expression. All outputs used a fixed JSON schema, enabling traceability and systematic evaluation (details are provided in Multimedia Appendix 1).

### AI Decision Aid

The AI decision aid is a multiagent conversational platform for antidepressant selection, serving as the system under evaluation in this black-box assessment. The system conducted structured intake through 6 sequential stages: establishing rapport, collecting illness history, gathering antidepressant history, documenting current medications, recording clinical procedures, and generating personalized recommendations. Each stage used LLM-guided dialogue to elicit clinical information and a Retrieval-Augmented Generation (RAG) system to normalize medical concepts before passing them to an analytical reasoning system for estimating antidepressant response. Concept normalization was a 2-step process: an embedding-based retriever returned the top-K candidate concepts from the study lexicon for each patient utterance, and an LLM selected the single best match from those candidates. Only this rank-1 selection was forwarded to the analytical model, and downstream candidates were discarded. If the correct concept was in the top K but not ranked first, it did not reach the recommendation step. The analytical advice system was based on the Direct Effects Multiplicative Inference (DEMI) algorithm [76], which estimates dependent Bayesian relationships among medical concepts to support clinical reasoning under partial observability. Alternative recommendation models (eg, logistic regression) are compatible, and the system specifications and code are publicly available [24].

### Evaluation Framework

#### Overview

The evaluation framework examined patient simulator performance across medical, linguistic, and behavioral dimensions: medical profiles through human and LLM-based annotation using a 3-label schema (accurate, inaccurate, and unsupported), and linguistic and behavioral profiles through human annotation, quantitative metrics, and visual clustering.

#### Human Annotation

All annotations were performed by 2 annotators with complementary domain expertise: one had an undergraduate degree in psychology, and one was a registered nurse. Each sample was independently annotated by both raters, with disagreements resolved through adjudication.

#### Medical Profile Evaluation

The patient simulator CoT process (1) identified relevant medical concepts, (2) rephrased each concept according to the linguistic profile, and (3) constructed a natural language response based on linguistic and behavioral characteristics. For each conversational turn, the simulator outputted (1) relevant concepts referenced numerically (eg, [2.3]) and (2) a natural language response with inline spans marking where each concept is expressed. These spans served as annotation units labeled using a three-category schema as follows: (1) *accurate*, where the medical fact is correctly expressed with minor colloquialisms (eg, “happy pills” for “Prozac”) permitted if core clinical meaning is preserved; (2) *inaccurate*, where the fact is present but misrepresented, distorting critical clinical details or using implausible phrasing; and (3) *unsupported*, where content does not correspond to the patient’s profile, capturing hallucinated or fabricated details outside tagged spans. This approach evaluated expression fidelity conditioned on correct concept retrieval. Concept recall was computed programmatically from the simulator’s traced outputs, with 95% of profile concepts appearing at least once in the generated conversations.

We validated the annotation and judging pipeline itself through controlled error injection. The patient simulator expressed nearly all medical concepts accurately, so annotators could achieve artificially high interannotator agreement (IAA) by labeling all concepts as *accurate*, and the LLM judge validation would lack discriminative power. To enable rigorous evaluation, we introduced controlled semantic perturbations by replacing clinical concepts with semantically similar but clinically distinct alternatives (eg, “hypertension,” “prehypertension,” “diabetes mellitus,” and “prediabetes”), selected via a semantic search over SNOMED CT and Current Procedural Terminology, 4th edition (CPT-4) that identified the top 20 candidates, which were randomly shuffled before ontology-based filtering removed hierarchical ancestors or descendants and enforced minimum hierarchical distance. The first qualifying candidate was selected, ensuring variation rather than defaulting to the nearest semantic neighbor; if none qualified, the threshold was relaxed or the pool expanded. The resulting perturbed profiles maintained linguistic realism while introducing subtle clinical inaccuracies for evaluating both human annotation quality and LLM judge performance.

#### Linguistic Profile Evaluation

Linguistic profiles were evaluated through human annotation, where annotators classified each conversation into 1 of 5 predefined linguistic profiles. Five automated metrics were also used:

1. Reading level: Flesch-Kincaid grade level (FKGL) [77] estimates the school grade level and is calculated per response turn and averaged per conversation.
2. Average response length: Average words per turn using NLTK’s English tokenizer [78].
3. Medical term density: Clinical term count via greedy n-gram matching (up to 6 grams) against the study medical lexicon (all concept codes present in the database).
4. Depression score: Mean turn-level probability from an XLM-RoBERTa–based depressive symptom classifier [79].
5. t-distributed stochastic neighbor embedding (t-SNE): Projects linguistic features to 2 dimensions using cosine distance; multiple seeds are tested to minimize Kullback-Leibler divergence.

#### Behavioral Profile Evaluation

Annotators classified each conversation into 1 of 3 behavior profiles. Three automated metrics quantified behavioral patterns:

1. On-topic similarity: Average cosine similarity between each AI decision aid turn and the patient simulator response.
2. Toxicity: Mean turn-level probability from the XLM-RoBERTa–based toxicity classifier [80].
3. t-SNE: Projects behavioral features analogously to linguistic t-SNE.

#### LLM Judge Evaluation

LLM-based judges have demonstrated strong performance in health care evaluation tasks [81-83]. Automated natural language processing similarity metrics, by contrast, have not been shown to correlate with expert human evaluation of generative LLM outputs on EHR data [84]. We used a separate LLM, Claude Opus 4.6, as an automated judge, applying the same annotation schema across medical, linguistic, and behavioral dimensions. The judge prompt was developed on 45 held-out conversations without human annotations. Judge alignment was then validated against human annotations on the full evaluation set, with results presented in the Results section.

### Experimental Paradigm

Experiments used 60 medical profiles evaluated across 4 antidepressants (sertraline: 17%, trazodone: 15%, fluoxetine: 13%, and duloxetine: 11%) reflecting common clinical practice [53]. For each antidepressant, a pool of patient profiles was simulated and down-sampled to 15 profiles per drug by stratifying across response probability bands. With *high*=7 and 3‐5 residual features per profile, generation yielded an average of 8‐9 clinical features per profile, consistent with reports that outpatient visits address 5.4‐7.1 clinical items per encounter [85]. This feature density reflects plausible patient disclosure and contrasts with All of Us records (approximately 98.88 features per patient).

The design comprised three settings: (1) 5 linguistic profiles with fixed *structured and cooperative* behavior (300 conversations); (2) 3 behavioral profiles with fixed *functional health literacy* (180 conversations); and (3) combined linguistic-behavioral variation (150 conversations), yielding 500 unique simulated conversations. The patient simulator and AI decision aid were powered by GPT-4.1. Claude Opus 4.6 served as the LLM judge, and all embedding-based analyses, including t-SNE projections and on-topic similarity, used OpenAI’s text-embedding-3-small model, which also supports RAG-based concept normalization in the AI decision aid. Both have been implemented as separate agents within LangGraph [86].

### Ethical Considerations

This project was examined by George Mason University’s Institutional Review Board (study 2154028‐1 for work on the All of Us database and study 00000437 for evaluation of the text of the advice through annotation) and was determined to be “not research” in the context of the definition of research on human subjects by the United States Department of Health and Human Services. Informed consent was therefore not required. All of Us data were accessed exclusively through the program’s secure Researcher Workbench under the data use agreement, and no individual-level records were redistributed. Annotation work was performed by named research personnel acknowledged in the manuscript. No human subjects were recruited for this study.

## Results

### Simulated Medical Profile Validation

Across 60 medical profiles, the framework demonstrated systematic risk characterization aligned with the NIST AI RMF Map and Measure functions. *Controllability* was achieved by emphasizing outcome-related features (37.2% of features per profile) while maintaining outcome diversity across response probability bands. *Coherence* was reflected in an average RR of 2.64 among outcome-related features, consistent with the RR gating. *Variability* was maintained through 292 unique concept codes across the 60 profiles. *Efficiency* was demonstrated by an average of 8.08 features per profile, substantially lower than the 98.88 features in a typical All of Us record. *Transparency* was maintained through explicit feature provenance across all generation components: demographic attributes followed All of Us distributions (age 13‐19 years: 748/515,405, 0.1%; 20‐40 years: 130,890/515,405, 25.3%; 41‐64 years: 213,726/515,405, 41.5%; 65‐79 years: 143,775/515,405, 28.0%; 80‐89 years: 26,266/515,405, 5.1%; male: 192,379/515,405, 37.3%; female: 323,026/515,405, 62.7%), outcome relevance was enforced using the top-K set, residual features were drawn from the non–top-K pool, and outcome probabilities matched antidepressant-specific binomial distributions (fluoxetine: range 0.28‐0.57, mean 0.41; sertraline: range 0.30‐0.59, mean 0.44; duloxetine: range 0.28‐0.57, mean 0.43; and trazodone: range 0.16‐0.42, mean 0.29).

### Medical Profile Validation

Medical profile fidelity was assessed through 1786 simulator-generated medical concepts with annotated spans across 100 conversations, including 292 perturbed concepts. Table 3 shows IAA between human annotators and the LLM judge. Across all concepts, human IAA was high (κ=0.73; *F*_1_-score=0.93). Agreement decreased for perturbed concepts (κ=0.30; *F*_1_-score=0.76), reflecting the difficulty of subtle semantic substitutions. Human-LLM judge agreement followed the same pattern (overall: κ=0.78; *F*_1_-score=0.93; perturbed concepts: κ=0.24; *F*_1_-score=0.77). Annotators identified 109 unique *unsupported* cases, predominantly incidental medication mentions (eg, Tylenol, ibuprofen, and vitamins) and minor symptom references (eg, headache and neck pain) not present in the medical profiles (IAA: *F*_1_-score=0.75). The LLM judge identified 76 *unsupported* cases of the same type (*F*_1_-score=0.58 against adjudicated human annotations). We also performed a paired bootstrap test [87] with 10,000 resamples. We found that human-human agreement (κ=0.73) was slightly higher than agreement between individual human annotators and the LLM (κ=0.70‐0.71), but these differences were not statistically significant (*P*=.21 and *P*=.32, respectively). Moreover, agreement between the LLM and each annotator did not differ significantly (*P*=.63), indicating no systematic annotator-specific bias. Overall, LLM-human agreement was comparable to human-human agreement within sampling variability.

**Table 3.** Agreement scores for human-human and human-LLM^a^ evaluations across full and perturbed concept sets.

Evaluation and concept set		Value, n	Cohen κ	Accurate *F*_1_-score	Inaccurate *F*_1_-score	Unsupported *F*_1_-score	Overall *F*_1_-score
Human-human							
Perturbed		292	0.30	0.45	0.85	—^b^	0.76
Full		1786	0.73	0.96	0.76	0.75	0.93
Human-LLM							
Perturbed		292	0.24	0.37	0.87	—^b^	0.77
Full		1786	0.78	0.97	0.81	0.58	0.93

a LLM: large language model.

b Not applicable.

In the LLM judge evaluation of medical profiles, of 8210 expressed concepts, 7932 (96.6%) were labeled *accurate*, 88 (1.1%) were labeled *inaccurate*, and 190 (2.3%) were labeled *unsupported*. The simulator maintained high accuracy (96%‐99%) and low error rates across all profiles. Within this range, concept expression frequency increased across the literacy gradient, peaking with *proficient*. Error types diverged modestly by profile: *proficient* showed the highest *unsupported* rate (10/981, 1.0%) due to greater medical term density, while *illness anxiety disorder* had the highest inaccuracy rate (30/1192, 2.5%) due to its repetitive tone. The *depression* profile produced the fewest expressed concepts with minimal errors.

Table 4 provides a breakdown of fidelity across linguistic and behavioral profiles. *Structured and cooperative* ensured stability, *distracted and unfocused* introduced frequent *unsupported* outputs, and *adversarial and combative* minimized errors, as confrontational responses tended to be terse, limiting opportunities for errors.

**Table 4.** LLM^a^ judge evaluation of medical profile fidelity across linguistic and behavioral profiles.

Profile name and metrics		Total, n	Accurate, n (%)	Inaccurate, n (%)	Unsupported, n (%)
Linguistic profiles under the structured and cooperative behavioral condition		4830	4724 (97.8)	66 (1.4)	40 (0.8)
Health literacy					
Limited		863	849 (98.4)	11 (1.3)	3 (0.3)
Functional		952	935 (98.2)	13 (1.4)	4 (0.4)
Proficient		981	968 (98.7)	3 (0.3)	10 (1.0)
Condition-specific					
Depression		842	826 (98.1)	9 (1.1)	7 (0.8)
Illness anxiety disorder		1192	1146 (96.1)	30 (2.5)	16 (1.3)
Behavioral profiles under the functional health literacy linguistic condition		3035	2906 (95.7)	18 (0.6)	111 (3.7)
Structured and cooperative		952	935 (98.2)	13 (1.4)	4 (0.4)
Distracted and unfocused		988	884 (89.5)	5 (0.5)	99 (10.0)
Adversarial and combative		1095	1087 (99.3)	0 (0.0)	8 (0.7)

a LLM: large language model.

The intersection of linguistic and behavioral profiles shaped error type rather than overall accuracy, which remained high across all combinations (Figure 3). *Distracted and unfocused* produced elevated *unsupported* rates in lower health literacy profiles, where conversational drift introduced off-profile content. *Adversarial and combative* drove inaccuracies in *proficient* and *illness anxiety disorder* profiles, where confrontational responses distorted rather than fabricated clinical content. *Structured and cooperative* yielded the lowest error rates, though *illness anxiety disorder* produced *unsupported* content even under cooperative conditions.

**Figure 3.**
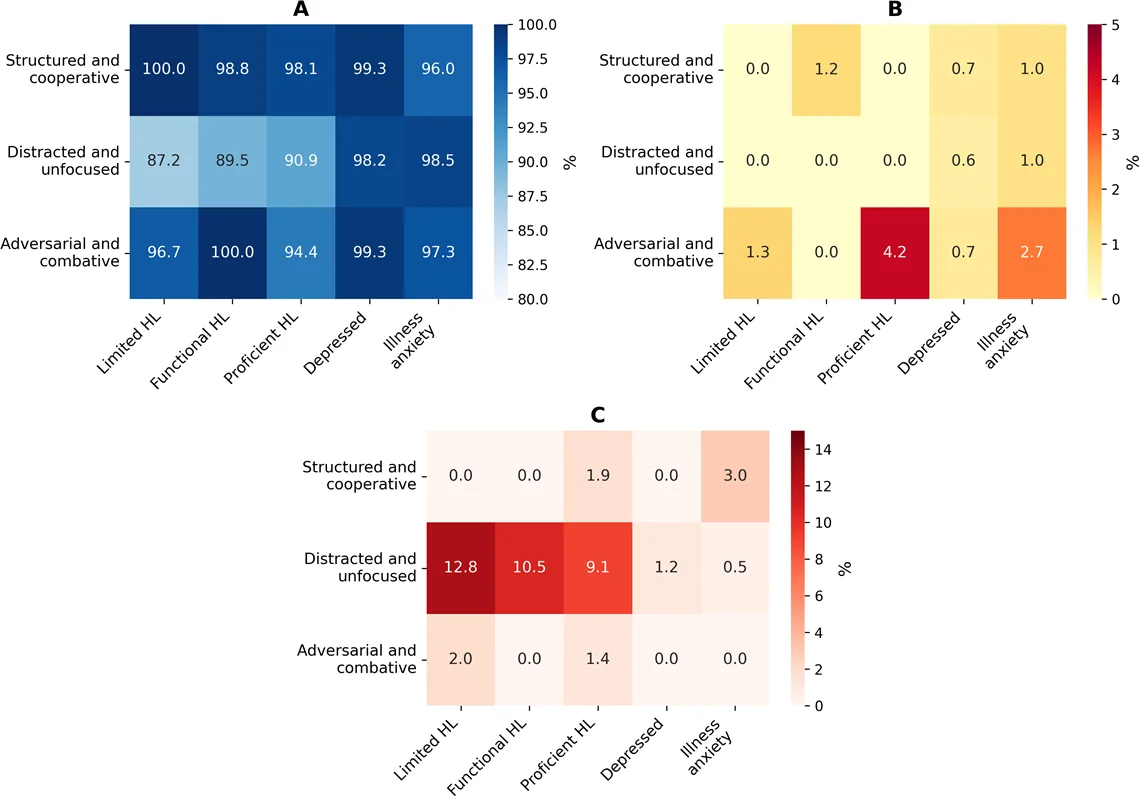
Large language model judge evaluations from the intersection of linguistic and behavioral profiles: (A) accurate, (B) inaccurate, and (C) unsupported. HL: health literacy.

### Linguistic Profile Validation

Human annotators showed substantial agreement on linguistic profile classification (κ=0.61; micro *F*_1_-score=0.70), comparable to human-LLM agreement (κ=0.63; micro *F*_1_-score=0.70). Adjudicated labels against predefined profiles reached a micro *F*_1_-score of 0.87, indicating consistent expression of intended profiles, and the LLM judge achieved a micro *F*_1_-score of 0.95 across 500 conversations.

Table 5 shows linguistic variation under fixed *structured and cooperative* behavior. Reading level increased monotonically from *limited* (FKGL=3.59) to *proficient* (FKGL=11.90), with response length and medical term density following the same gradient (*limited*: 34.34 words, 4.33 medical terms; *proficient*: 38.91 words, 11.68 medical terms). The *depression* profile produced the shortest responses (21.48 words; depression score=0.20), whereas *illness anxiety disorder* produced the longest responses (65.05 words; medical term density=14.27; depression score=0.25), reflecting greater health-related elaboration.

**Table 5.** Evaluation of linguistic profiles under the structured and cooperative behavioral condition.

Profile name and metrics		Reading level (FKGL^a^)	Response length	Medical term density	Depression score
Health literacy					
Limited		3.59	34.34	4.33	0.01
Functional		7.63	28.70	9.83	0.00
Proficient		11.90	38.91	11.68	0.00
Condition-specific					
Depression		4.49	21.48	6.17	0.20
Illness anxiety disorder		8.11	65.05	14.27	0.25

a FKGL: Flesch-Kincaid grade level.

Figure 4 shows t-SNE clustering where linguistic profiles form distinct clusters, with *functional* and *proficient* overlapping due to their shared characteristics, and *depression* partially overlapping with lower literacy profiles. Conversely, *illness anxiety disorder* formed a distinct cluster. Together, these findings confirm graded, distinct linguistic profiles.

**Figure 4.**
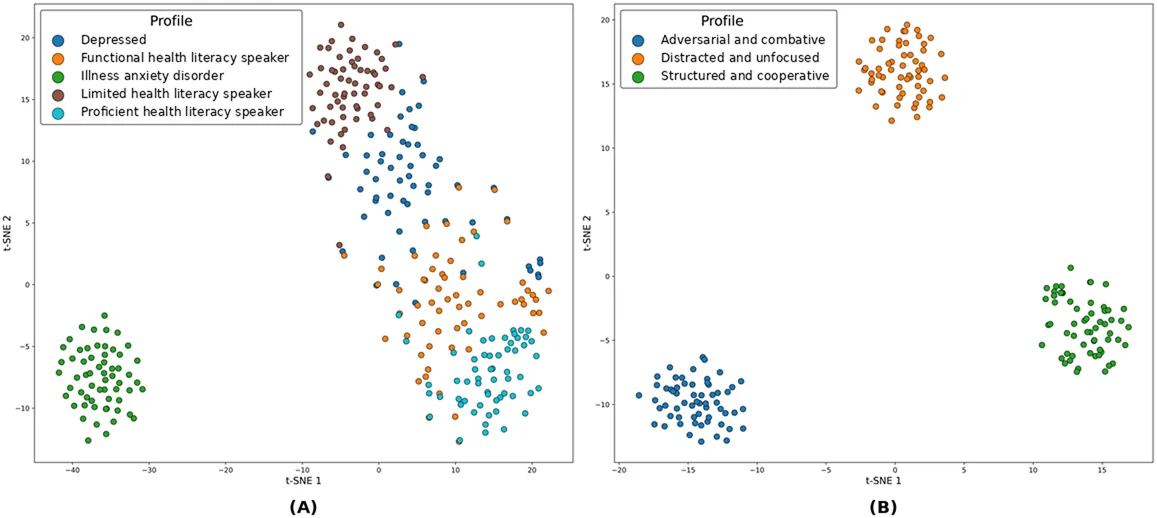
t-distributed stochastic neighbor embedding visualization of response embeddings for linguistic profiles under the structured and cooperative behavioral condition (A) and behavioral profiles under the functional health literacy linguistic condition (B).

### Behavioral Profile Validation

Human annotators showed high agreement on behavioral profile classification (κ=0.93; micro *F*_1_-score=0.96), identical to human-LLM agreement, with classification against predefined profiles reaching a micro *F*_1_-score of 0.98. Under fixed *functional health literacy* (Table 6), *structured and cooperative* achieved baseline on-topic similarity (0.51) and the lowest toxicity (0.0003). *Distracted and unfocused* showed slightly reduced on-topic similarity (0.50), consistent with conversational drift, while maintaining low toxicity (0.0035). *Adversarial and combative* exhibited substantially higher toxicity (0.0564) while retaining the highest topical relevance (0.52).

**Table 6.** Evaluation of behavioral profiles under the functional health literacy linguistic condition.

Profile	On-topic similarity	Toxicity
Structured and cooperative	0.51	0.0003
Distracted and unfocused	0.50	0.0035
Adversarial and combative	0.52	0.0564

Figure 4 shows t-SNE clustering with clear separation among behavioral profiles. Together, these findings confirm distinct behavioral profiles under fixed linguistic conditions.

### Profile Interactions

Figure 5 presents intersectional evaluation across linguistic and behavioral profile combinations. *Distracted and unfocused* produced the longest responses across all linguistic profiles (61‐82 words vs 21‐66 for *structured and cooperative*), while *depression* consistently produced the shortest responses (21‐32 words) with elevated depression scores (0.18‐0.25). Toxicity showed the strongest behavioral dominance, with *adversarial and combative* exhibiting elevated toxicity (range: 0.03‐0.11) regardless of linguistic profile, whereas reading level and depression scores remained largely determined by linguistic profiles. On-topic similarity showed modest behavioral effects, with *structured and cooperative* achieving slightly higher alignment than *distracted and unfocused*. These patterns demonstrate that the simulator maintains profile independence where intended (linguistic features preserved across behaviors) while capturing realistic interactions (behavioral effects on engagement and tone).

**Figure 5.**
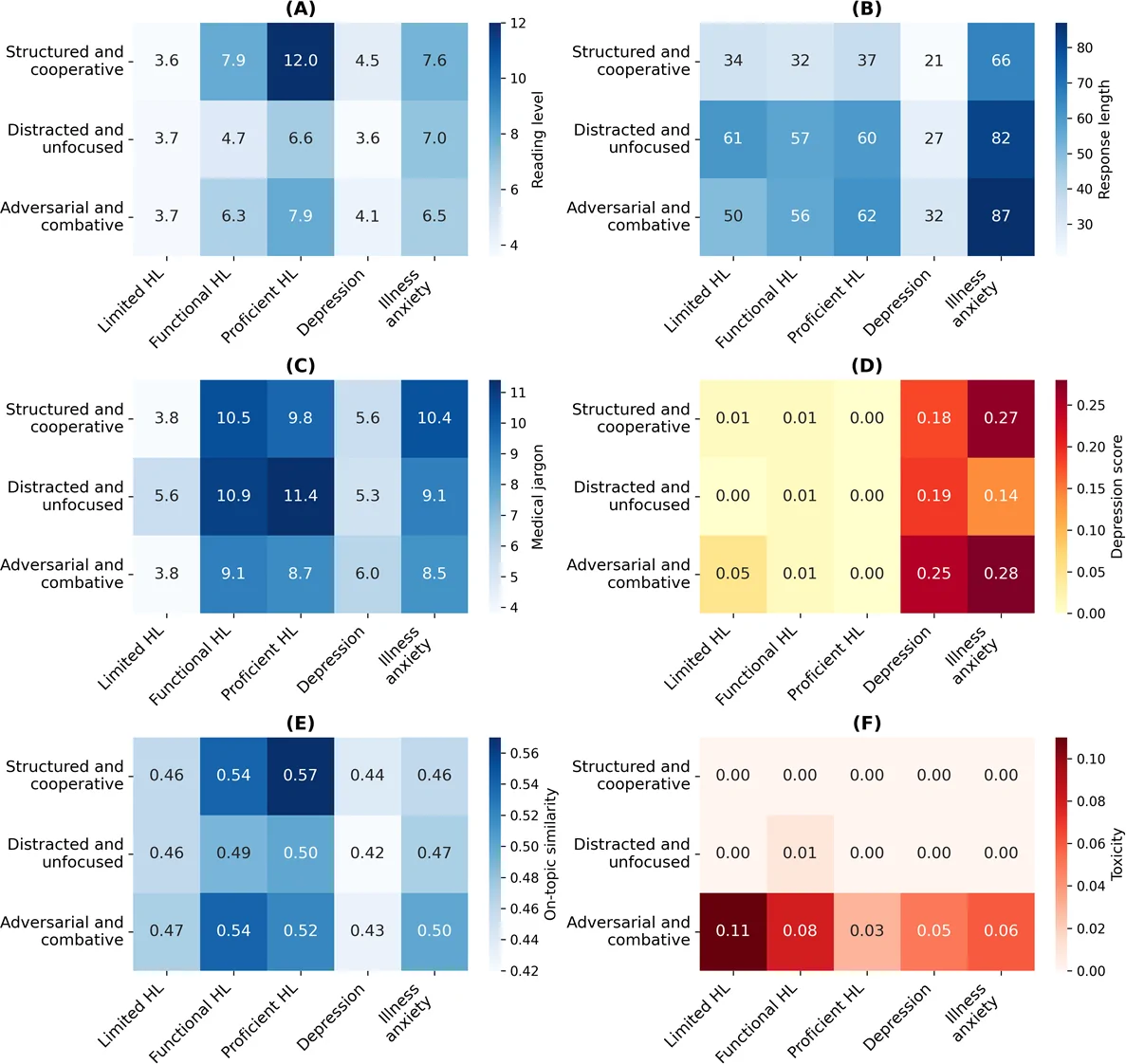
Intersection of linguistic and behavioral profiles across evaluation metrics: (A) reading level, (B) response length, (C) medical jargon, (D) depression score, (E) on-topic similarity, and (F) toxicity. HL: health literacy.

### AI Decision Aid Performance: Risk Measurement Across Patient Variation

#### Overview

This section evaluates AI decision aid performance across 500 simulated conversations.

#### Concept Retrieval Coverage

Overall concept recall reached 93% (n=2622) across 2819 reference concepts, indicating that approximately 7% (n=197) were not identified by the AI decision aid. Recall was the highest for diagnoses (1958/2077, 94.3%) and procedures (518/569, 91.0%), while medications showed the lowest recall (146/173, 84.4%), identifying medication retrieval as a relative vulnerability. The system additionally introduced 151 concepts outside predefined profiles, predominantly medications (n=84) and procedures (n=57), reflecting conversational elaboration.

#### Retrieval Accuracy and Health Literacy Effects

The retrieval performance of concepts identified by the AI decision aid was reported on the full set of simulator-generated reference concepts (n=2819), with unidentified concepts contributing to overall outcomes. Overall rank-1 accuracy was 65.9% (1857/2819); however, 80.2% (2261/2819) of concepts appeared within the top 20 candidates, indicating ranking imprecision rather than missing semantic coverage. Retrieval accuracy increased with health literacy level. Rank-1 accuracy ranged from 47.6% (*limited*) to 81.9% (*proficient*). Condition-specific profiles fell in between, with *depression* at 62.0% and *illness anxiety disorder* at 63.7%. Vague or affect-heavy language reduced ranking accuracy (Multimedia Appendix 3).

Patients with *limited* health literacy or *depression* used colloquial, fragmented, or affect-heavy language that misaligned with the system’s canonical concept vocabulary. Because top-20 retrieval did not fully close this gap, the system introduces a structural bias: retrieval is reliable for patients who use precise medical terminology but degrades for populations expressing equivalent clinical content through less structured language (Multimedia Appendix 3).

#### Downstream Risk to Antidepressant Recommendation

Recommendations were produced by DEMI over 15 antidepressants plus a *no recommendation* category (probability <0.1), making downstream performance sensitive to information loss during intake. Performance improved with higher health literacy, with *proficient* achieving the highest *F*_1_-score (0.73) under s*tructured and cooperative* and *limited* achieving the lowest *F*_1_-score (0.48). Behavioral profiles further modulated this risk: *structured and cooperative* yielded more stable outcomes, while *distracted and unfocused* and *adversarial and combative* were associated with performance shifts that varied by linguistic profile, as shown in Figure 6.

**Figure 6.**
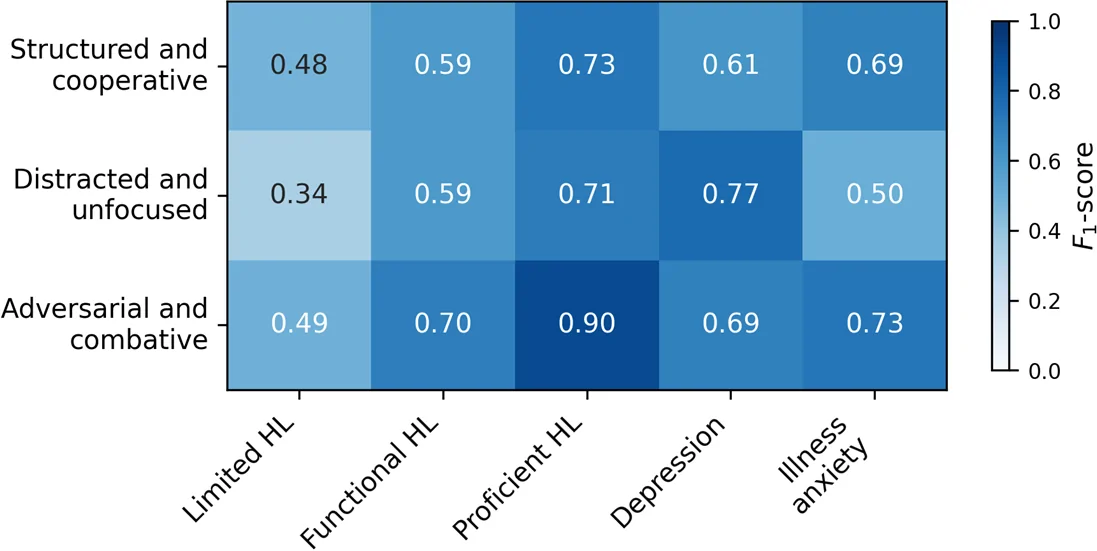
*F*_1_-scores for antidepressant recommendation before and after AI decision aid processing across linguistic and behavioral profile combinations. HL: health literacy.

## Discussion

### Overview

This study demonstrated that patient simulation, grounded in the NIST AI RMF, can systematically expose risks in conversational health care AI that static evaluation methods miss. Across 500 simulated conversations, the framework revealed a monotonic performance gradient across health literacy levels, with corresponding effects on antidepressant recommendation accuracy. The remainder of this section discusses these principal findings, their clinical implications, and the limitations of the work.

### Principal Findings

#### Medical Profile Generation

The medical profile generation process produced clinically coherent profiles with explicit feature lineage traceable to source EHR data. To validate both annotator and LLM judge performance, we applied controlled error injection. Semantically similar but clinically distinct perturbations (eg, hypertension → prehypertension, diabetes mellitus → prediabetes, major depressive disorder moderate → major depressive disorder mild, and generalized anxiety disorder → adjustment disorder with anxiety) tested annotator and LLM judge sensitivity. The injection was challenging by design. Each perturbation is semantically close to the original concept, the kind of subtle substitution an LLM is likely to produce, and once rendered in a patient’s communication style, it is easily mistaken for an accurate expression. Agreement on perturbed concepts was lower than overall agreement (human-human: κ=0.30 vs κ=0.73; human-LLM: κ=0.24 vs κ=0.78). This partly reflects the intended difficulty of the perturbations, which approximate real-world concept ambiguity, but also signals a real detection limit relevant to deployment monitoring. If human raters and the LLM judge both struggle to catch subtle clinical substitutions under these controlled conditions, comparable errors are unlikely to be easier to catch in live use, where similar ambiguity could go undetected without dedicated monitoring.

#### Linguistic and Behavioral Profile Effectiveness

The simulator produced measurably distinct profiles across both dimensions. Behavioral profiles formed categorically different clusters, while linguistic profiles formed a continuous health literacy gradient. The *functional* and *proficient* overlap reflects their genuine similarity along this continuum. Cross-dimensional analysis confirmed profile independence with 2 interaction effects: *distracted and unfocused* consistently increased response length, and *adversarial and combative* increased toxicity regardless of linguistic profile.

#### Health Literacy as a Risk Factor for Clinical AI

The linguistic profile substantially affected AI decision aid performance, with monotonic degradation across the health literacy gradient: rank-1 retrieval ranged from 47.6% (*limited*) to 69.6% (*functional*) and to 81.9% (*proficient*), with the gradient persisting in downstream antidepressant recommendation accuracy. The system relied on precise terminology: colloquial expressions like “my morning pill for my nerves” force the retrieval system to resolve a larger semantic distance than “20 mg of fluoxetine for generalized anxiety disorder.” These failures point to specific Manage-function interventions: clarification prompting, terminology normalization, or multipass retrieval. Condition-specific profiles showed intermediate retrieval performance (*depression*: 62.0%, *illness anxiety disorder*: 63.7%), suggesting that affective expression also degrades performance, though less severely than low health literacy.

#### LLM Judge Viability

The LLM judge aligned strongly with human annotators on medical concept agreement (overall *F*_1_-score=0.93) and showed lower agreement on perturbed concepts (*F*_1_-score=0.77), mirroring divergence among human annotators on those same items. The judge also flagged *unsupported* content beyond the perturbations, related to incidental medications and minor symptoms not present in the profiles. These results support LLM judge use for large-scale screening, with human review reserved for edge cases.

### Comparison With Prior Work

Patient simulators have advanced rapidly, yet none of these simulators have jointly addressed medical, linguistic, and behavioral risks within a single risk-aligned framework. PATIENT-Ψ [47] achieves strong behavioral and emotional realism through 106 expert-authored cognitive behavioral therapy cognitive schemas, with a focus on clinical training rather than EHR-grounded risk assessment. PatientSim [35] benchmarks LLM doctor agents using MIMIC-derived clinical profiles and a 4-axis persona space (personality, language proficiency, recall, and confusion), with persona treated as a fixed categorical attribute and traceability provided at the persona level rather than the concept level. MATRIX [57] audits hazards across 2100 dialogues using a safety-engineered hazard taxonomy, a simulated patient (PatBot), and an LLM judge, with clinical content drawn from scenario templates and a self-contained safety taxonomy rather than alignment to an external risk-management framework. Cook et al [34] demonstrated training scalability through prompt-engineered GPT virtual patients on 2 outpatient topics, with the evaluation directed at clinician history-taking rather than at the AI system itself. In contrast, our patient simulator unifies 3 risk-aligned profile dimensions in a single architecture: EHR-grounded medical profiles drawn from All of Us via RR gating, 5 linguistic profiles spanning 3 health literacy levels (*limited*, *functional*, and *proficient*) and 2 condition-specific styles (*depression* and *illness anxiety disorder*), and 3 behavioral profiles (*structured and cooperative*, *distracted and unfocused*, and *adversarial and combative*). The framework aligns with the NIST AI RMF Map and Measure functions and provides concept-level lineage from each utterance back to its source clinical facts.

### Clinical Implications

#### Overview

Performance differences across health literacy and behavioral profiles raise clinical, equity, and governance concerns that should be addressed before the AI decision aid is deployed.

#### Health Literacy as a Patient Safety Issue

Rank-1 retrieval fell from 81.9% (*proficient*) to 47.6% (*limited*). Because antidepressant trials typically last 6-12 weeks before effectiveness can be assessed [88,89], retrieval errors may not surface until after weeks of ineffective or harmful treatment. *Limited health literacy* is more common among patients with severe depression, lower socioeconomic status, advanced age, and less formal education [90,91], which are the same groups that bear the heaviest burden of untreated mental illness [92,93]. The performance gap is concentrated in the population most in need of accessible support. This is not a technical limitation to be addressed in a later release; it is an immediate patient safety risk requiring active mitigation before deployment.

#### Behavioral Profiles Reflect Psychiatric Phenotypes

The *distracted and unfocused* profile mirrors the psychomotor slowing, impaired attention, and disorganized thought of moderate-to-severe depression [94,95], which are core symptoms of the illness rather than mere variations in communication style. The *adversarial and combative* profile often reflects trauma history, prior negative health care experiences, or treatment frustration rather than a difficult personality [96,97]. The system is least reliable for patients whose illness severity itself makes communication difficult. These clinical interpretations explain why the observed performance gaps carry safety significance; the behavioral profiles were derived from a general interaction taxonomy [74] and have not been validated against symptom presentations aligned with the Diagnostic and Statistical Manual of Mental Disorders (DSM). Thus, the mapping to specific psychiatric phenotypes should be read as illustrative rather than diagnostic.

#### Care Pathway Integration Determines Harm Potential

Provider-supervised use makes the identified gaps manageable, and direct-to-patient deployment, particularly to underserved populations with limited provider access, amplifies them. Deployment plans should specify the oversight model, review criteria, and escalation pathways for low-confidence recommendations. Under the NIST AI RMF, these governance structures correspond directly to Manage-function commitments and should be specified before deployment approval. Where the system informs treatment selection, providers should also interpret its outputs as patterns of medication persistence, recognizing that persistence and symptom remission can diverge in real-world treatment, with prognostic implications [98]. The Manage-function interventions (clarification prompting, terminology normalization, and adaptive retrieval) should be developed with input from patients with limited health literacy and providers experienced in psychiatric communication, and validated in the same simulation framework before rollout.

#### Equity and Participatory Design

The performance gradient documented here is a familiar pattern in health care AI. Tools designed and validated on data representing dominant communication styles reinforce existing disparities at deployment scale rather than reducing them [99]. Antidepressant selection compounds this risk. Comorbidity, pharmacogenomics, and social determinants all shape outcomes, and patients with limited health literacy already face diagnostic and treatment gaps in standard psychiatric care [100,101]. A system that underperforms for these populations risks widening those gaps, and technical correction alone will not address this. The identified Manage-function interventions should be co-designed with patients carrying limited health literacy and with clinicians who serve them. Psychiatric care has developed practices for this work, including teach-back protocols, plain language standards, and culturally adapted patient education [102]. Conversational AI for these populations should be built on the same foundation.

### Limitations

#### Generalizability

Simulated conversations have not been validated against real patient interactions. Ecological validity will be evaluated in a planned real-world trial. The evaluation covers a single clinical task and 4 antidepressants. The framework itself does not assume a deployment model, but the harm potential of the performance gaps we report depends on how the AI decision aid is deployed. Future evaluations should match the oversight model under which it will be used.

#### Validity of Clinical Signals

Antidepressant response was operationalized as 10-week medication persistence without switching or augmentation, a surrogate influenced by formulary access, provider inertia, and barriers to follow-up, which does not capture clinical remission [103,104]. Future work should anchor response in patient-reported outcomes or validated symptom scales (eg, Patient Health Questionnaire-9 and Hamilton Rating Scale for Depression) [105,106].

#### Profile Construction

Medical profiles relied exclusively on structured EHR data, omitting the unstructured clinical narrative where clinicians document symptom severity, treatment history, and psychosocial context. The independence screening criterion may exclude valid coupled comorbidities, underrepresenting patients with strongly correlated conditions. Behavioral profiles follow the taxonomy by Simpson et al [74] and are not validated against DSM-aligned presentations: the *distracted and unfocused* profile resembles depressive neurovegetative symptoms but is not clinically verified, and the *adversarial and combative* profile does not distinguish oppositional engagement from trauma- or frustration-driven presentations. Validation against psychiatrically characterized populations remains a priority.

#### Model Dependence

The patient simulator and AI decision aid both used GPT-4.1, though information leakage between them was structurally prevented. The simulator’s internal profile and concept markers were stripped before its response reached the AI decision aid, which extracted clinical information from natural language text alone, as it would from a real patient. A shared model family could still produce correlated blind spots not present with 2 independently trained systems. The LLM judge (Claude Opus 4.6) was a different model, mitigating this risk at the evaluation layer but not for the simulator-decision aid pairing. Future work should test decision aid performance against simulator output using an alternative model family on a subsample.

#### Annotation Diversity

All annotations were performed by 2 raters. While their complementary clinical backgrounds (psychology and nursing) and adjudicated agreement provide some assurance, a 2-rater design limits the generalizability of the reported agreement estimates and cannot capture the full range of expert interpretation. Future evaluations should incorporate a larger, more diverse annotator pool.

### Conclusions

Applied to a conversational antidepressant decision aid, our patient simulation framework found a steep gap in performance across health literacy levels. Rank-1 concept retrieval was 81.9% for proficient health literacy and 47.6% for limited literacy. Recommendation accuracy followed the same pattern. This gap is important, as limited health literacy is common among patients with the most severe psychiatric illness. The patients least served by this system are those who need it the most. Identifying this risk required simulated patients grounded across all 3 dimensions: medical, linguistic, and behavioral. Prior simulators have varied 1 or 2 of these dimensions, but not all 3 together. While we evaluated the framework only on antidepressant selection, extending it to other clinical decision-aid tasks remains a direction for future work. The primary contributions include a RR-based algorithm for auditable medical profile generation, a controlled perturbation method for validating annotators, and an LLM judge with human-level agreement. Scalable simulation and evaluation are also enabled, and thus, candidate mitigations can be developed and tested against the same risks before deployment. However, technical fixes for AI systems are not sufficient. Health systems must specify care pathways, oversight protocols, and escalation requirements. Moreover, they must clinically validate the system in the populations it currently underserves.

## Supplementary material

10.2196/100772 Multimedia Appendix 1 Patient simulator.

10.2196/100772 Multimedia Appendix 2 Medical and behavioral profiles.

10.2196/100772 Multimedia Appendix 3 Retrieval performance.

## Abbreviations

AI RMF: AI Risk Management Framework
CoT: chain-of-thought
CPT-4: Current Procedural Terminology, 4th edition
DEMI: Direct Effects Multiplicative Inference
DSM: Diagnostic and Statistical Manual of Mental Disorders
EHR: electronic health record
FKGL: Flesch-Kincaid grade level
GAN: Generative Adversarial Network
IAA: interannotator agreement
LIWC: Linguistic Inquiry and Word Count
LLM: large language model
NIST: National Institute of Standards and Technology
PCORI: Patient-Centered Outcomes Research Institute
RAG: Retrieval-Augmented Generation
RQ: research question
RR: risk ratio
SNOMED CT: Systematized Nomenclature of Medicine Clinical Terms
t-SNE: t-distributed stochastic neighbor embedding
