# Supplementary material for: A Patient Simulation Framework for Risk Assessment of Conversational Health Care AI: Development and Evaluation Study
Source: JMIR AI. 2026 Oct 5;5:e100772. doi: 10.2196/100772 (PMC13638073; doi:10.2196/100772)
Supplement: Supplementary file 2 [file ai-v5-e100772-s002.docx]

# Multimedia Appendix 2: Medical and Behavioral Profiles

## Medical Profiles

**Phase – 1: Medical Profile Generation**

This section presents the medical profile generation procedure used by the analytical advice system in detail. The method follows a staged selection strategy that balances outcome relevance, statistical coherence, and controlled diversity to produce clinically plausible and informative profiles.

Outcome Relevance (Top-K Filter): The algorithm first restricts the candidate space to the Top‑K predictors of the antidepressant response outcome e₀ (here, K = 500). This filter improves sample efficiency and signal‑to‑noise by focusing subsequent selection on variables with demonstrated individual association to e₀. As in classical filter style feature selection, an initial relevance screen improves downstream generalization and reduces variance in later steps. In spirit, this aligns with the “max‑relevance” component of minimum‑redundancy‑maximum‑relevance (mRMR), which explicitly prioritizes features that are informative about the target before addressing redundancy.

Coherence and Approximate Independence via a Symmetric Risk‑Ratio Gate: New candidates are screened using a symmetric acceptance band around unity based on aggregated pairwise risk ratios with respect to the already‑selected set S. Let $RR(s, v)$ denote the risk ratio between an existing event $s \in S$ and a candidate event $v$, and let ${aggregate}_{s\in S}$ be a conservative aggregator (e.g., MAX) over these pairwise values. Acceptance condition:

$$1/1.5<{aggregate}_{s\in S} RR\left( s,v \right)\leq high$$

This symmetric band $(low = 1/1.5)$ respects the multiplicative interpretation of $RR$ treating protective associations $(< 1)$ and harmful associations $(> 1)$ in a balanced manner while capping near deterministic couplings $(> high)$ that would introduce redundancy or multicollinearity. Excluding values $\ll1$ also avoids strongly anticorrelated additions that can create unstable compensations in downstream predictions [1]. Using MAX as the aggregator is conservative (rejects $v$ if it couples too strongly with any $s \in S$). Controlled Diversity from the Residual Event Pool: After establishing the coherent core of relevant events (Top-K features), the algorithm introduces controlled diversity by exploring the residual event pool. Those features not included among the top-ranked relevant set. In this phase, a residual candidate $u \in R$ is admitted only if it exhibits a meaningful positive association with at least one of the already selected relevant features $s \in S$. This step aims to capture latent or contextually related variables that were not strongly ranked individually but still contribute to plausible patient variability when correlated with the existing core.

To ensure statistical coherence, independence is defined as the range $1/1.5 \leq RR(s,v) \leq1.5$; any relationship within this interval is treated as neutral and thus ignored. Accordingly, only candidates showing stronger associations i.e., $RR(s,v) > 1.5$ or $RR(s,v) < 1/1.5$ are eligible for inclusion, provided they were not part of the original relevant set. Diversity condition:

$$\exists s \in S : RR(s,v) > 1.5$$

where R denotes the residual pool (features outside the Top-500), and S represents the previously selected relevant features. This mechanism ensures that the resulting simulated patient profiles include a bounded degree of heterogeneity driven by correlated but previously under-ranked features, enriching diversity without compromising coherence [1,2]

The Risk Ratio categories and the corresponding feature inclusion logic are illustrated in Figure S1, which depicts the transition from negatively associated to positively associated features and their mapping across Step 3 (relevant) and Step 4 (coherent but irrelevant) feature sets.

Figure S1: Graphical representation of Risk Ratio thresholds and feature selection workflow.

The medical profile generation algorithm constructs a simulated clinical history by selecting events that are both clinically meaningful and statistically consistent with real world response patterns. The process begins with a set of variables that strongly predict response to the target antidepressant, then filters these candidates through risk based constraints to ensure independence and realistic variability. A second expansion stage introduces additional events that provide controlled diversity without creating improbable clinical combinations. The final set of events forms a structured medical profile that reflects both individual level variability and population level statistical associations.

The inputs listed below define the core elements required for the generation of each simulated medical profile. They specify the clinical target, the size of the simulated population, the available concept codes, and the demographic distributions from which each patient begins, along with the parameters that control risk ratio constraints and diversity expansion.

Inputs:

- e0 – Concept code of response of target antidepressant
- N– Number of patients to simulate
- All_Concept_Code – List of all the concept code
- G – Categorical distribution over gender (At Birth) codes
- A – Categorical distribution over age-bin codes
- high – Maximum allowed Risk Ratio for selecting a variable
- max_residual_additions – Limit on how many residual-pool events may be appended

The helper routines provide the key decision checks used throughout the selection process. They evaluate the compatibility of candidate events with the partially constructed profile and ensure that all additions remain within the required risk ratio bounds. These routines allow the algorithm to enforce statistical constraints without interrupting the main flow of the pseudocode.

**Helper Routines:**

- $PassesRRGate(selected,candidate\_event,low,high)$ - Returns $TRUE$ if the aggregated pairwise risk ratio $RR(s,candidate\_event)$ across all s in selected satisfies $1/1.5 < aggregate \leq high$. Otherwise returns $FALSE$.
- $AnyRRExceeds(selected,candidate\_event,threshold)$ - Returns $TRUE$ if there exists at least one event s in selected such that $RR(s,candidate\_event)$ exceeds the given threshold. Otherwise returns $FALSE$.
- $PredictResp(S,e₀)$: Returns the probability of response to the antidepressant associated with concept $e₀$, given the selected event set $S$, computed using the DEMI algorithm.

Figure S2 combines the inputs and helper routines into a four-stage algorithm that builds each medical profile step by step. It begins with a Top-K predictor filter and initializes demographic seeds, then applies an independence screen governed by high, the upper similarity threshold used in the RR gate, to ensure statistical coherence, and finally introduces controlled diversity through residual events capped by max_residual_additions, which can be randomly drawn from a range (e.g., 3-5) rather than fixed. Together, these stages create a clinically plausible and statistically grounded event set for each simulated patient, with high and max_residual_additions offering two tunable levers for controlling profile density: lowering high narrows the set of eligible candidates, while max_residual_additions directly sets how many additional features are added.

**Procedure:**

**# Step‑1: Selection of Top-K and Initialization**

1. V ← Top500_Predictor_of_e₀(e₀).	\\ Top_500 predictors

2. R ← {All_Concept_Code} \ {V ∪ G ∪ A}	\\ Residual Event

3. Initialize output container T ← ∅.

4. For each patient j = 1..N:

5.	S ← ∅	// selected set.

6.	Aᵥ ← V; Aᵣ ← R	// fresh available pools.

**# Step‑2: Demographics Seed**

7.	S ← S ∪ {weighted_random_choice(G)}	\\ gender

8.	S ← S ∪ {weighted_random_choice(A)}	\\ age-bin

**# Step‑3: Independence-Screened Selection**

9.	While Aᵥ ≠ ∅:

```
10.		v ← random_choice(Aᵥ).
11.		If PassesRRGate(S, v, high=high, low=1/1.5):
```

12.	Set S ← S ∪ {v}.

```
13.		Remove v from Aᵥ.
14.	Define S_intermediate ← S.
```

**# Step‑4: Controlled Diversity Expansion**

15.	i ← 1.

16.	While Aᵣ ≠ ∅ and i ≤ max_residual_additions:

```
17,		 u ← random_choice(Aᵣ).
18.		If AnyRRExceeds(S_intermediate, u, threshold = 1.5):
```

19.	Set S ← S ∪ {u}; i ← i + 1.

20.	Remove u from Aᵣ.

21.	Compute final p̂ ← PredictResp(S, e₀).

22.	Append (j, e₀, |S|, S, p̂) to T.

Figure S2. Medical profile generation algorithm

**Phase – 2: Probabilistic Patient Selection Using Binomial Distribution**

The second phase of the medical profile generation process selects a subset of simulated patients whose response probabilities collectively reproduce the population-level response distribution observed in the reference dataset. Each simulated patient from Phase 1 is associated with a predicted probability of antidepressant response between 0 and 1. The selection aims to retain a cohort whose aggregate distribution mirrors the expected binomial pattern defined by the population mean response rate ($p$) and total number of patients ($n$).

To operationalize this, the probability range [0, 1] is divided into seven probability bands corresponding to approximate σ-intervals of a binomial distribution $B(n, p)$. Each simulated patient is assigned to a band according to their predicted probability of response. The population mean ($\mu= np$) and standard deviation ($\sigma= \surd(np(1 - p))$ ) define the expected frequency of patients in each band, which acts as a target allocation weight. For example, for $n = 100$ and $p = 0.4$, the binomial distribution has $\mu= 40$ and $\sigma\approx4.9$; approximately $64 \%$of the population lies within $(\mu- \sigma, \mu+ \sigma]$, $15 \%$ on each side within ($\mu\pm1\sigma to \mu\pm2\sigma$], and only a few percent beyond $\mu\pm2\sigma$. Table S1 shows a generalized sigma-band allocation for the binomial distribution $B(n, p),$ including the corresponding ranges and their interpretations.

Table S1: Generalized Sigma-Band Allocation for Binomial Distribution $B (n, p)$.

| **Sigma Band Interval** | **Range of Success Counts (k)** | **Approximate Probability Mass (%)** | **Interpretation** |
| --- | --- | --- | --- |
| k ≤ μ − 3σ | 0 ≤ k ≤ ⌊μ − 3σ⌋ | ≈ 0.1–0.3 % | Extremely low outcome region |
| (μ − 3σ, μ − 2σ] | ⌊μ − 3σ⌋ + 1 ≤ k ≤ ⌊μ − 2σ⌋ | ≈ 2–3 % | Moderately below average region |
| (μ − 2σ, μ − 1σ] | ⌊μ − 2σ⌋ + 1 ≤ k ≤ ⌊μ − 1σ⌋ | ≈ 14–16 % | Slightly below average region |
| (μ − 1σ, μ + 1σ] | ⌊μ − 1σ⌋ + 1 ≤ k ≤ ⌊μ + 1σ⌋ | ≈ 63–65 % | Central or typical outcome region |
| (μ + 1σ, μ + 2σ] | ⌊μ + 1σ⌋ + 1 ≤ k ≤ ⌊μ + 2σ⌋ | ≈ 14–16 % | Slightly above average region |
| (μ + 2σ, μ + 3σ] | ⌊μ + 2σ⌋ + 1 ≤ k ≤ ⌊μ + 3σ⌋ | ≈ 2–3 % | Moderately high outcome region |
| k > μ + 3σ | ⌊μ + 3σ⌋ + 1 ≤ k ≤ n | ≈ 0.1–0.3 % | Extremely high outcome region |

Patients are then sampled proportionally to these weights so that the empirical distribution of predicted response probabilities in the final cohort approximates the theoretical binomial distribution. This approach ensures that the selected subset preserves both the central tendency and natural variability of the population while avoiding over- or under-representation of extreme responders. The outcome is a statistically balanced and demographically diverse cohort suitable for downstream simulation and evaluation.

## Behavioral Profiles

We present the mapping of 13 commonly observed patient behaviors documented by Simpson et al. [3] into four higher-level behavioral categories*:* *Inquisitive and Open-Ended,* *Reserved & Minimalist*, *Distracted & Unfocused*, and *Adversarial & Combative* in Table S2. In addition to these four categories, *Structured & Cooperative* is included as the baseline behavioral category.

Table S2: Mapping of behavioral profiles to their associated patient behaviors.

| **Behavioral Profile** | **Associated Patient Situation** |
| --- | --- |
| Inquisitive & Open Ended | Is uncertain what they want |
| Reserved & Minimalist | Is reluctant to work together Is anxious, worried, or upset Worries they will never get better |
| Adversarial & Combative | Is angry Is demanding Brings up negative feelings in the clinician |
| Distracted & Unfocused | Is difficult to direct or disorganized Has an unclear diagnosis Is an unreliable historian Has a difficult time changing their behavior Does not take medications regularly Has difficulty planning ahead |

## References

1.	Peng H, Long F, Ding C. Feature selection based on mutual information criteria of max-dependency, max-relevance, and min-redundancy. *IEEE Trans Pattern Anal Machine Intell*. 2005;27(8):1226-1238. doi:10.1109/TPAMI.2005.159

2.	Guyon I, Elisseeff A. An introduction to variable and feature selection. *Journal of machine learning research*. 2003;3(2003):1157-1182.

3.	Simpson S, McDowell A. *The Clinical Interview: Skills for More Effective Patient Encounters*. Routledge; 2019. doi:10.4324/9780429437243
