# Supplementary material for: A Patient Simulation Framework for Risk Assessment of Conversational Health Care AI: Development and Evaluation Study
Source: JMIR AI. 2026 Oct 5;5:e100772. doi: 10.2196/100772 (PMC13638073; doi:10.2196/100772)
Supplement: Supplementary file 3 [file ai-v5-e100772-s003.docx]

# Multimedia Appendix 3: Retrieval Performance

Table S1 details retrieval performance by linguistic profile over all reference concepts.

Table S1: *AI Decision Aid* Retrieval Performance for Identified Concepts (Reported over All Reference Concepts)

| **Linguistic Profile** |  | **n** | **Rank-1** | **Top-20** | **Not Retrieved** | **Mean Rank (non-Rank-1)** |
| --- | --- | --- | --- | --- | --- | --- |
| **Health Literacy** | Limited | 460 | 47.6% | 71.5% | 12.0% | 14.5 |
|  | Functional | 1023 | 69.6% | 83.1% | 8.8% | 8.5 |
|  | Proficient | 453 | 81.9% | 85.2% | 10.2% | 9.2 |
| **Condition-specific** | Depression | 440 | 62.0% | 79.5% | 9.8% | 9.6 |
|  | Illness Anxiety Disorder | 443 | 63.7% | 77.9% | 9.9% | 13.0 |
|  | **Overall** | **2,819** | **65.9%** | **80.2%** | **9.9%** | **11.2** |

Figure S1 breaks down Rank-1 and Rank-20 accuracy across linguistic profiles, behavioral profiles, and their intersections. The health literacy gradient is visible across all behavioral conditions, with *Limited* health literacy consistently producing the lowest Rank-1 accuracy and the widest gap between Rank-1 and Rank-20, indicating that correct concepts are retrieved but fail to rank first.

Figure S1: Rank-1 and Rank-20 accuracy for linguistic profiles, behavioral profiles, and their intersections.
